# Supplementary material for: Turning to religion as a mediator of the relationship between hopelessness and job satisfaction during the COVID-19 pandemic among individuals representing the uniformed services or working in professions of public trust in Poland
Source: PLoS One. 2023 Dec 7;18(12):e0291196. doi: 10.1371/journal.pone.0291196 (PMC10703260; doi:10.1371/journal.pone.0291196)
Supplement: Attachment — Submitted filename: Responses.pdf [file pone.0291196.s002.pdf]

Dear Reviewer 1,

Thank you very much for the Reviewer's careful reading of our manuscript. The suggested changes have improved the manuscript considerably. We hope that you will find our manuscript acceptable in its current form. All the improvements introduced are presented below.

Point 1: I thank the authors provided a detailed description of the COR Theory, which will be useful for reader unfamiliar with its principles. However, since the COR developed over the years, I would add also a recent reference, such as for example Hobfoll et al., 2018, doi: 10.1146/annurev-orgpsych-032117-104640

Response 1: We have added the indicated reference to the manuscript and bibliography.

Point 2. I thank the authors for including a detailed description but, again, I think that a citation would provide a useful link for further readings.

Response 2: We have added the publication given below to the manuscript and bibliography.

McIntosh CN, Fischer DG. Beck's cognitive triad: One versus three factors. Canadian Journal of Behavioural Science. 2000;32(3): 153–157. doi.org/10.1037/h0087110.

Point 3. I thank the authors for correcting the results reported in the paper. However, I think that a double check would still be necessary. (6.1) For example, results in Figure 3 are internally consistent, given that the sum of direct and indirect effect in the lower part (−0.351*0.102−0.245) are equal to the total effect in the upper part (−0.281), as well as with the correlation in Table 2 (again, −0.281). However, to my understanding, the standardized beta of hopelessness on turning to religion in Figure 3 (−0.351) should be equal to a bivariate correlation coefficient, which is however -0.171 in Table 2.

Response 3: We do appreciate the Reviever's careful reading and analyzing our manuscript. We do agree with the Reviewer. Thanks to the Reviewer's meticulousness we could correct the mistake made in the table. We have made the corrections.

Point 4. Second, as far as I understand, standardized regression coefficients are reported in Figure 3, but unstandardized regression coefficients are shown in Figure 4, which makes results difficult to compare. What is the rationale behind this choice?

Response 4: The coefficients in Figure 3 and in the text have been changed into non-standardized ones. We have made it to ensure uniformity. Moreover, we have used the recommendations made in the publication: Fairchild AJ, McQuillin SD. Evaluating mediation

and moderation effects in school psychology: a presentation of methods and review of current practice. J Sch Psychol. 2010 Feb;48(1):53-84. doi: 10.1016/j.jsp.2009.09.001. PMID: 20006988; PMCID: PMC5488867.

Point 5: Similarly, the unstandardized regression coefficient of hopelessness on turning to religion in Figure 4 (−0.600) does not seem consistent with the standardized regression coefficient in Figure 3 (−0.351), but please correct me if I am wrong. (6.4) Finally, please also note that the symbols "-" and "−" are interchangeably used for "minus". Similarly, both "." and "," are used as decimal separator (e.g., Figure 3).

Response 5: We have made the corrections.

Point 6. I thank the authors for the thorough discussion of possible limitations concerning the cross-sectional research design. However, I do not understand the reference to the "Monte Carlo Power Analysis for Indirect Effects application developed by Schoemann, Boulton and Short". As far I understand, this procedure is aimed at investigating statistical power, not the presence of common method bias. In this respect, please see a review by Podsakoff et al., 2003 (doi: 10.1037/0021-9010.88.5.879).

Response 6: We are grateful for the literature suggested. We do agree with the Reviewer that this procedure is aimed at investigating statistical power. The reference to the application developed by Schoemann, Boulton and Short is inappropriate and does not provide any useful information. We apologize for the confusion.

Point 7: Finally, I noticed some typos, so the manuscript needs a revision of the English form.

Response 7: The manuscript has been revised by a native speaker.

Dear Reviewer 2,

We are grateful for the Reviewer's positive evaluation of our work and valuable suggestions that, in our view, helped us to improve the manuscript.
